# Changes in lipid droplets morphometric features in mammary epithelial cells upon exposure to non-esterified free fatty acids compared with VLDL

**DOI:** 10.1371/journal.pone.0209565

**Published:** 2018-12-31

**Authors:** Ronit Mesilati-Stahy, Nurit Argov-Argaman

**Affiliations:** The Department of Animal Science, The Robert H. Smith Faculty of Agriculture, Food and Environment, Rehovot, The Hebrew University of Jerusalem Israel; University of Illinois, UNITED STATES

## Abstract

The effects of the macrostructure of long chain fatty acids on the lipid metabolism and biosynthesis of lipid droplets (LD) was studied in mammary epithelial cells (MEC). MEC were exposed to similar compositions and concentrations of fatty acids in the form of either triglycerides (Tg), as part of the very-low-density lipids (VLDL) isolated from lactating cow plasma, or as non-esterified- free fatty acids (FFA). Exposing MEC to FFA resulted in two distinct processes; each independently could increase LD size: an elevation in Tg production and alterations in phospholipid (PL) composition. In particular, the lower PC/PE ratio in the FFA treatment indicated membrane destabilization, which was concomitant with the biosynthesis of larger LD. In addition, 6 fold increase in the cellular concentration of the exogenously added linoleic acid (C18:2) was found in MEC treated with FFA, implying that long chain fatty acids administrated as FFA have higher availability to MEC, enabling greater PL synthesis, more material for the LD envelope, thereby enhancing LD formation. Availability of long chain fatty acids administrated as VLDL-Tg, is dependent on LPL which its activity can be inhibited by the hydrolysis products. Therefore, we used increasing concentrations of albumin, to reduce the allosteric inhibition on LPL by the hydrolysis products. Indeed, a combined treatment of VLDL and albumin, increased LD size and number, similar to the phenotype found in the FFA treatment. These results reveal the role played by the macrostructure of long chain fatty acids in the regulation of LD size in MEC which determine the size of the secreted MFG.

## Introduction

Milk fat is secreted in a structure termed milk fat globules (MFG) which consists of a triglyceride (Tg) core covered with three layers of polar lipids (PL) and proteins derived from the mammary gland epithelial cellular membranes [1]. MFG are secreted in a wide range of sizes, from less than 200 nm to over 15 μm [1] and their size is closely associated with their fatty acids (FA), PL and protein composition [2] [3] with higher PL content in smaller MFG. Thus, MFG size plays an important role in regulating milk composition. MFG size regulation is still elusive, although understanding the underlying mechanisms could provide a tool to control milk composition, which has nutritional value as well as industrial implications: smaller MFG have higher content of PL, a desirable bioactive component which was demonstrated to improve cognitive and physiological development in infants and health attributes in adults [4–10]. The importance of understanding MFG size regulation is also relevant for the dairy industry as MFG size affects organoleptic properties of dairy products; it can modulate cheese ripening, structure, as well as shelf life of dairy products [11–13]. Previously it was demonstrated that MFG size can be modulated through diet and the size differences affect dairy products properties. For example, reduction in size and increased specific surface area of the MFG yielded softer and more crumbly cheeses [14–15]. In addition, improved spread ability of cream and butter was received when processing milk with smaller MFG produced by dairy cows fed diet supplemented with fish meal [16].

Despite its direct effect on milk organoleptic properties and human health, MFG size regulation has scarcely been studied and therefore, the mechanisms regulating this unique feature of milk are still elusive. MFG size is determined by the size of its precursors, the intracellular lipid droplets (LD) synthesized in the mammary epithelial cells (MEC; [17–19]). The initial steps of intracellular LD synthesis and its structure are common to various cell types, including, but not limited to muscle cells, adipocytes and hepatocytes [20]. The common synthesis process involves initial formation of Tg droplet between the endoplasmic reticulum (ER) membranes which is release into the cytoplasm covered with one layer of PL and specific surface-associated proteins originating from the ER [17–18, 21–23].

From their site of synthesis, the cytoplasmic LD migrates toward the apical pole of the cell, where they are apocrine-secreted into the alveolar lumen, enveloped by the apical cell membrane bilayer. Eventually, the secreted MFG consists of a Tg core covered with three layers of PL and proteins, derived from the cellular membranes, termed milk fat globule membrane (MFGM) [19].

PL plays a central role in regulating LD size, by either controlling membrane stability and hence the susceptibility of LD to fusion, or by providing the coating material for the Tg core [24–25]. Membrane stability is regulated mainly by phosphatidylcholine (PC) and phosphatidyethanolamine (PE), and the ratio between them [24]. PC, a cylinder like PL, have been suggested to stabilize the LD surface and inhibit fusion, while PE, a negatively curved cone-like PL, destabilizes the LD surface which enhance fusion events and therefore induce the formation of larger LD [24]. In a wider cellular context, PC and PE are connected in an enzymatic chain reaction which can convert PC to phosphatidylserine (PS) via the phosphatidylserine synthase 1 (PSS1) pathway in the endoplasmic reticulum and its mitochondrion-associated membrane. Then PS can be converted to PE by the action of phosphatidylserine decarboxylase (PSD) in the mitochondria, and back to PC through the phosphatidylethanolamine N-methyltransferase (PEMT) pathway [26]. These reactions can modulate the balance between PE and PC content in cellular membranes and change membrane stability. Interestingly, PE synthesis by PSD occurs in the mitochondria [26]. Therefore, the mechanism controlling membrane stability and hence fusion between LD may be subjected to metabolic pathways and signals that regulate mitochondrial activity or number in the cell [27–28].

In lipogenic tissues, long chain fatty acids have a role in activating PPARs family of transcription factors and their co-activators PGC-1 α and β which together regulate the expression of mitochondrial enzymes and transport proteins, thus regulating mitochondrial activity [29]. In MEC, long-chain fatty acids are absorbed as preformed fatty acids from the capillary bed surrounding the mammary gland alveolar lumen [30–34]. The preformed fatty acids can originate from the diet, circulating as very-low-density lipoproteins (VLDL) [35–36]. Alternatively, preformed long chain fatty acids may originate from adipose lipolysis, circulating as non-esterified, free fatty acids (FFA). The dominant macro-structure of long-chain FA circulating in blood (i.e. FFA or VLDL) which are available for utilization by peripheral tissues including MEC, depends on the animal’s metabolic status [37–41].

Long chain fatty acids from VLDL are available to peripheral tissues, including the mammary gland, through either VLDL receptor endocytosis [34] or the lipoprotein lipase (LPL) pathway [42]. On the other hand, FFA can enter the cell by fatty acid-binding protein (FABP) mediation [43]. FABP forms complex with CD36 and facilitate the transport of fatty acids through the membrane, directing them to the cellular location where Tg or PL synthesis takes place [44].

This implies that fatty acids macro-structure (i.e. FFA or VLDL), is relevant in terms of bioavailability of preformed fatty acids to the cells and to PL synthesis, and thus may affect MFG size. Moreover, the source of the FFA either from VLDL hydrolysis or from non-esterified FFA induced different metabolic response in the cell as was shown by Ruby et al. [45] who demonstrated activation of peroxisome proliferator-activated receptor (PPAR) α upon exposure to VLDL-hydrolysis products and not by FFA [44]. Therefore, the structure of long chain fatty acid available to cells may influence metabolic and molecular pathways which may be involved in mitochondria biogenesis and activity, factors which are much relevant to MEC productivity and to the regulation of MFG size.

The hypothesis that the macrostructure of long chain fatty acids may affect LD size is supported by findings in hepatocytes, demonstrating that elevated levels of FFA are associated with large cytoplasmic LD and greater cellular content of Tg [29]. More specifically, in the mammary gland stage of lactation is negatively correlated with non-esterified FFA content in blood and MFG size, [46–47].

Taken together, these results imply that FFA has a role in the mechanisms underlying the regulation of MFG size; however the mechanism is still unknown. In vivo, this association is difficult to study because milk yield and total milk fat concentration, as well as homeorasis signals are all strong confounders of the relationship between MFG size and FFA in plasma.

Accordingly, the aim of the present study was to isolate the effects of the biochemical macrostructure of preformed, long chain fatty acids (in the form of FFA vs. VLDL) on the morphometric features of MFG precursors, the MEC intracellular LD.

## Materials and methods

### Materials

DMEM/F12, penicillin, streptomycin, amphotericin B, L-glutamine solution, trypsin–EDTA solution C and fetal bovine serum (FBS) were all obtained from Biological Industries (Beit Haemek, Israel). Bovine insulin, hydrocortisone, ovine prolactin, bovine serum albumin (BSA) solution, hyaluronidase and DNase I were purchased from Sigma Aldrich Israel Ltd. (Rehovot, Israel). Collagenase type II was purchased from Worthington Biochemical Corporation (Lakewood, NJ, USA).

### VLDL isolation

Blood samples were collected from the coccygeal vein of Israeli Holstein lactating cow, using vacuum tubes containing lithium heparin (BD, Plymouth, UK. The procedures used in this study were approved by the Volcani Center Animal Care Committee (Ethical clearance number- IL 610/15). The experiment was conducted at the Volcani Center experimental farm in Bet Dagan, Israel. The tubes were immediately placed on ice and centrifuged at 3,000*g* for 15 min at room temperature. Plasma was collected and ultracentrifuged in a salt solution (11.42 g NaCl/liter) in a density of 1.006 g/ml [48]. Briefly, 16 ml of plasma was overlaid on 8 ml salt solution and centrifuged at 45,000 rpm for 20 h at 17°C in a Sorvall t-865 rotor. Supernatant was collected as VLDL (1 ml) and its lipid and fatty acids composition was determined by high-performance liquid chromatography (HPLC) and GC, respectively. The fatty acids composition and concentration was used to reconstruct a mix of free acyl chains (FFA) that mimicked the composition and concentration of the VLDL fatty acids.

### Primary culture preparation

MEC for primary culture were isolated from cow mammary gland according to a protocol established in our laboratory [28]. Study protocols were in compliance with the regulations of the Israeli Ministry of Health, under the supervision of the Department for control of Animal products, State of Israel Ministry of Agriculture rural development veterinary services and animal health. Certificate Nu: # 80. Briefly, udder tissue was collected from three lactating cows at Haifa commercial slaughterhouse (32694 IL). Tissue were collected after cows were commercially sacrificed by certified worker of the slaughterhouse and transferred to the laboratory. Tissues were immediately submerged in DMEM/F12 medium supplemented with 10% (w/v) FBS, 100 U/ml penicillin, 100 μg/ml streptomycin, 0.25 μg/ml amphotericin B, 1 μg/ml insulin and 0.5 μg/ml hydrocortisone (growth medium). Tissue (10 g) was digested by shaking in 100 ml of growth medium supplemented with collagenase (1mg/ml), hyaluronidase (1mg/ml) and heparin (0.02 mg/ml) at 100 rpm for 3 h at 37 °C. After incubation, the suspension was filtered through a metal mesh (250 μm) and the filtrate was centrifuged at 350 *g* for 5 min. The sediment was treated with trypsin–EDTA and 0.04% (w/v) DNase. The cells were then washed with growth medium supplemented with heparin and treated with DNase alone, filtered through a 100-μm cell strainer (BD Falcon, Bedford, MA) and washed with the growth medium.

Primary MEC Cells were plated at 150,000 cells per 60-mm plastic dish for cellular lipid extraction and RNA extraction, or at 50,000 cells per well in 6-well plates on glass coverslips for Nile red or Mitotracker staining. After overnight incubation, the medium was replaced with DMEM/F12 without serum, containing 0.15% (w/v) FFA-free BSA and insulin (1 μg/ml), hydrocortisone (0.5 μg/ml) and prolactin (1 μg/ml) for 48 h to induce milk lipid and protein synthesis. Then cells were treated with FFA or VLDL for 24 h in the presence of insulin (1 μg/ml), hydrocortisone (0.5 μg/ml) and prolactin (1 μg/ml).

### Treatments

Four fatty acids were used for the FFA treatment, based on the VLDL composition: palmitic (19% w/v), stearic (23%, w/v), oleic (8%, w/v) and linoleic (50%, w/v), diluted with DMEM/F12 medium supplemented with 0.5% FFA-free BSA to a final concentration of 100 μM, which based on our established protocol induced changes in metabolic status of the cells and was not toxic [27–28]. For the VLDL treatment, we used at the same concentration of BSA. When applicable, albumin was added to the VLDL-treated cells at increasing concentrations (0.5 mg/ml (as in the ctrl), 15 mg/ml and 31.86 mg/ml).

### LD staining

Cells grown on glass coverslips were rinsed with PBS, fixed with 4% paraformaldehyde in PBS and stained with Nile red (200 nM, Sigma, St. Louis, MO, USA). Coverslips were then rinsed 3 times with PBS and stained with DAPI (Sigma) for 5 min. The coverslips were rinsed again with PBS and mounted with fluorescent mounting medium (Dako, North America Inc., Carpinteria, CA, USA).

### Determination of mitochondrion quantity

Cells were incubated in DMEM/F12 medium with 250 nM Mitotracker deep red FM stain (Cell Signaling Technology) for 30 min at 37°C. The cells were then fixed in ice cold 100% methanol for 15 min at -20°C and rinsed three times with PBS. Cells were mounted with fluorescent mounting medium (Dako) and slides were visualized with an Olympus DP73 digital camera using CellSens Entry software version 1.7 (Olympus). Mitochondrial fluorescence in each cell was quantified by ImageJ software version 1.48 (Bethesda, MD, USA) using the following formula: corrected total cell fluorescence = integrated density of selected cell—(area of selected cell x mean fluorescence of background readings).

### Fluorescence microscopy and LD Size measurements

Slides were visualized with an Olympus BX40 fluorescence microscope equipped with an Olympus DP73 digital camera using CellSens Entry software(version 1.7, Olympus), and with an Olympus IX81 confocal microscope using Fluoview 500 software. LD diameter was measured using ImageJ software version 1.48. LD were divided into two or three (for the albumin supplementation experiment) size groups: <1 μm, between 1 and 2 μm, and >2 μm. LD larger than 1 μm were designated “large” and those smaller than 1 μm were designated “small”.

### Lipid extraction and analysis

#### Chemicals and reagents

For lipid extraction, methanol and chloroform (both analytical reagent grade) were purchased from Bio-Lab Ltd. (Jerusalem, Israel). For HPLC analysis, chloroform and ethanol (used at 97:3 v/v, both analytical reagent grade) and methanol (HPLC grade) were purchased from Bio-Lab. The triglyceride triolein (>99% pure) was purchased from Supelco (Bellefonte, PA). The PL standards were supplied by Sigma Aldrich Ltd. Israel and consisted of: PE (1,2-dioleoyl-sn-glycero-3 phosphoethanolamine, 10 mg PL per mL CHCl_3_, purity 99%), PI (L-α phosphatidylinositol ammonium salt, from bovine liver, purity 98%), PS (1,2-dioleoyl-sn-glycerol-3-phospho-L-serine sodium salt, purity 95%), PC (1,2-dioleoyl-sn-glycero-3-phosphocholine, purity ≥99%) and SM (sphingomyelin; from bovine brain, purity 97%). Conjugated linoleic acid (cis-9 trans-11 linoleic acid, purity >95%) was used as a standard for FFA. For the GC analysis, methanol (analytical reagent grade) was purchased from Bio-Lab, petroleum ether (analytical reagent grade) was from Gadot Lab Supplies (Netanya, Israel) and sulfuric acid was from Bet Dekel (Ra’anana, Israel). Retention times were determined by injection of commercial standard mixes of PL and Tg.

### Sample collection and lipid extraction

#### VLDL lipid extraction

Total lipids were extracted from VLDL using a protocol adapted from the cold-extraction procedure developed by Folch et al. [49]. Total lipids were extracted from 0.5 ml VLDL with methanol–chloroform–water (1:2:0.6, v/v). After overnight incubation at 4°C, the upper phase was removed and the lower phase was collected and filtered through a 0.45-μm Teflon syringe filter (Axiva Sachem Biotech, Ahmadabad, India) into a new vial. The solvent was then evaporated under vacuum, and 100 μl chloroform–ethanol (3%, v/v) was added. Total extracted lipids were stored at -20°C until further analysis. For GC analysis, fatty acids methyl esters (FAME) were generated by incubation of the lipid extract with 2.5 ml of 5% (v/v) H_2_SO_4_–methanol at 65°C for 1 h in sealed vials. After incubation, the lipid phase was extracted with petroleum ether and the solvent was evaporated under vacuum. FAME were dissolved in a final volume of 100 μl petroleum ether before injection for GC analysis.

#### Cell lipid extraction

At the end of the treatments, cells were harvested with trypsin, washed with 0.9% NaCl and stored at -20°C until lipid extraction. Total lipids were extracted from the cells using a protocol adapted from the cold-extraction procedure developed by Folch et al. [35]. Each sample was extracted twice, once for lipid analysis by normal-phase LC and once for fatty acids analysis by GC. Total lipids were extracted from 0.5 ml VLDL or 0.25 ml cells in PBS with methanol–chloroform–water (1:2:0.6, v/v).

After overnight incubation at 4°C, the upper phase was discarded and the lower phase was filtered through a Pasteur pipette with glass wool. Samples were then evaporated under a nitrogen stream at 65°C, diluted in 100 μl chloroform−methanol (3%, v/v) and stored at -20°C for HPLC analysis. For GC analysis, FAME were generated by methyl esterification and dissolved in a final volume of 100 μl petroleum ether before injection for GC analysis.

### GC analysis

To determine VLDL fatty acids composition and cell composition after the treatments, lipid extraction and methylation were performed on VLDL isolated from cow blood and on the harvested cells. Chromatographic analysis was performed with a GC (Agilent Technologies, Santa Clara, CA, USA) equipped with a fused-silica capillary column (60 m × 0.25 mm ID, DB-23, Agilent Technologies) under the following conditions: the oven temperature was programmed from 130°C to 170°C at a rate of 27°C/min, from 170°C to 215°C at a rate of 2°C/min, held at 215°C for 8 min, from 215°C to 250°C at a rate of 40°C/min, held at 250°C for 5 min. Run time was 37.9 min. Helium was used as the carrier gas at 2.21 ml/min. Flame ionization detector temperature was 270°C, and injector temperature was 280°C. Air and hydrogen flows were adjusted to give maximal detector response. Peak identification was based on relative retention times of two external standards. For polyunsaturated fatty acids (PUFA), PUFA-2 (Sigma Aldrich Israel Ltd, Rehovot, Israel), and a FAME C8:0 to C24:0 mix (Supelco, Bellefonte, PA, USA). The area of each fatty acid peak was recorded using ChemStation software (Agilent Technologies). Fatty acids were recorded as percentage of total fatty acids within each sample (mol%).

### Analysis of polar and neutral lipids

Separation of polar and neutral lipids was performed by HPLC (HP 1200, Agilent Technologies) combined with an evaporative light-scattering detector (1200 series ELSD, Agilent Technologies). The separation protocol was conducted with a gradient of dichloromethane, 99% methanol and 1% ammonium, and double-distilled water according to a previously described protocol [2] using normal-phase chromatography on a silica column (Zorbax RX-SIL, 4.6 × 250 mm, Agilent Technologies). Briefly, the column was heated to 40°C, flow was 1 ml/min, and injection volume was 20 μl. The ELSD was heated to 65°C, nitrogen pressure was 3.8 bars, filter was 5, gain (sensitivity) was set to 4 for the first 14 min, then changed to 12 until 21 min, and then changed to 7 until the end of the run, to enable detection of differently abundant lipid components. The separation process was managed by Chem Station software (Agilent Technologies), which permitted the acquisition of data from the ELSD detector. Identification and quantification of the separated lipids were performed using external standards (Sigma Aldrich). Quantification was performed by external standard curves and expressed as amount per 10^6^ live cells. Live cell number was determined after Trypan blue staining, using a hemocytometer. Membrane composition is presented as weight percentage of each PL out of the summed quantity of recognized PL in each sample [2].

### Gene-expression analysis

#### RNA extraction and cDNA synthesis

Total RNA was isolated from primary culture MEC by Gene Elute Mammalian Total RNA Miniprep Kit (Sigma Aldrich) according to the manufacturer’s instructions. The concentration and 260/280 nm optical density ratio of the RNA was determined by Nanodrop spectrophotometer (NanoDrop Technologies, Wilmington, DE, USA). RNA samples were kept at -80°C until further analysis. Total RNA (1 μg) was reverse-transcribed to produce cDNA using the qScript cDNA Synthesis Kit (Quanta Biosciences) according to the manufacturer’s instructions.

#### Real-time PCR analysis

Primers were designed by Primer-BLAST software (NCBI, http://www.ncbi.nlm.nih.gov/tools/primer-blast/index) based on cDNA sequences published in the National Center of Biotechnology Information database or taken from the literature, as indicated (Table 1). Primers were synthesized by Sigma Aldrich Israel Ltd. (Rehovot, Israel). cDNA was mixed with primers and platinum SYBR Green qPCR Supermix-UDG without ROX (Invitrogen Corporation, Carlsbad, CA, USA). Mx3000P Real-Time PCR System (Stratagene, La Jolla, CA, USA) was used. Analysis was performed by MxPro software version 4.10 (Stratagene). Dissociation-curve analysis was performed after each real-time experiment to confirm the presence of only one product. The efficiency of the reaction and the initial mRNA quantity in the sample were determined using LightCycler 96 software version 1.1 (Roche, Basel, Switzerland), and the ΔΔCt method was used to calculate the relative expression of each gene. Data were normalized to the geometrical mean of two housekeeping genes: *UXT* and *β2-microglobulin*.

**Table 1:** Primer sequences used for Real-time PCR analysis.

Gene	Accession number	Sequence (5'→3')	Size (bp)	Reference
DGAT-1	NM_174693.2	F: CGACTCCTGGAGATGCTGTT	116	Self-designed
		R: ATGCGGGAGTAGTCCATGTC		
α-Lactalbumin	NM_174378.2	F: TGTCTCTCGCTCCTGGTAGG	106	Self-designed
		R: ACCTCCGTAGCCCTTCAAGT		
LPL	NM_001075120.1	F: CCACATGCCCTACTGGTTTC	106	Self-designed
		R:AGTCGCCTTTCTCCTGATGA		
PPAR-γ	NM_181024.2	F: CAGTGTCTGCAAGGACCTCA	110	Self-designed
		R:GATGTCAAAGGCATGGGAGT		
UXT	BQ676558	F: TGTGGCCCTTGGATATGGTT	101	Bionaz & Loor, 2007
		R: GGTTGTCGCTGAGCTCTGTG		
2β microglobulin	NM_173893	F:CATCCAGCGTCCTCCAAAGAT	131	Harvatine & Bauman, 2006
		R: CCCCATTCTTCAGCAAATCG		
PEMT	NM_182989.3	F: TTCTGGAATGTGGTTGCGAGA	116	Cohen et al., 2015
		R:AGGACGTTCAAGAGCAGGATG		
Ndufaf3	NM_001046105.2	F: ACGAGCTGTATCAACGGACG	162	Cohen et al., 2015
		R: AACCTACGTTCCACTGCACC		
FABP	NM_001078162.2	F: CCTTATCCGCCGCTTTATC	91	Self-designed
		R: TCTCCGTCAGCTTCCAGGTA		

### Statistical analysis

All statistical procedures were performed using JMP software (version 12; SAS Institute, Cary, NC, USA). All data are reported as means ± SEM. Treatment and experiment number were defined as fixed effects in the model. All dependent variables were checked for homogenic variance by unequal variances in JMP software and where variance was not homogenic, Welch’s ANOVA test was performed. Comparisons were made by ANOVA followed by Tukey–Kramer HSD test.

## Results

### Altered LD size phenotype upon exposure to FFA vs. VLDL

**Fig 1:**
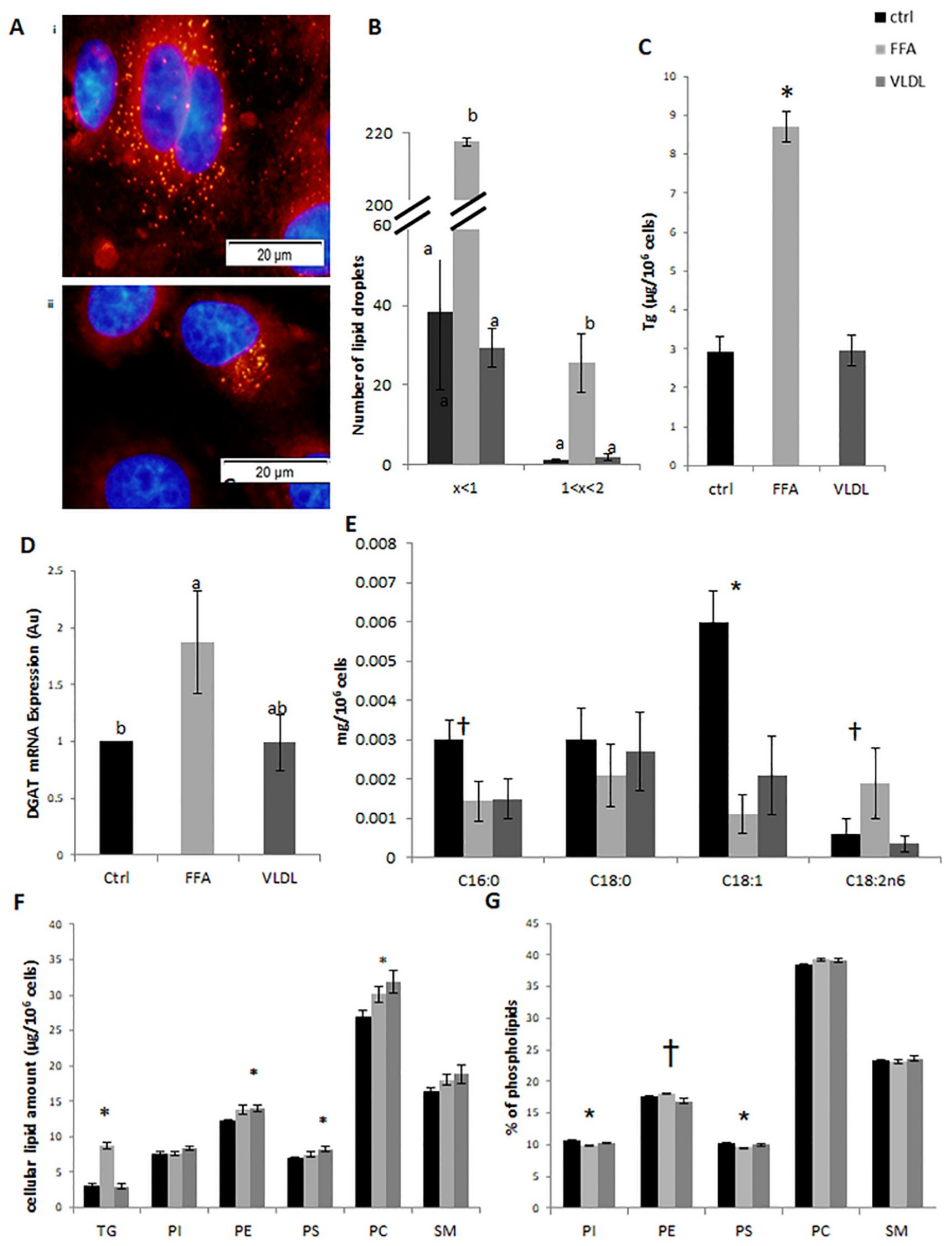
Long chain fatty acid macro structure change LD size and membrane phospholipid composition in a different manner. Mammary epithelial cells (MEC) were treated with 100 μM very-low-density lipoprotein (VLDL) or 100 μM free fatty acids (FFA) for 24 h. (A) MEC nuclei was stained with DAPI (blue) and neutral lipids were stained with Nile red (red). LD were more numerous and larger under the FFA treatment (i, top) compared to VLDL (ii, bottom). Scale bars, 20 μm. (B) Number of LD by size category (>1 μm, between 1 and 2 μm) in control (black), FFA (light gray) and VLDL (dark gray) treatments, n = 4 for each replicate in each treatment, total of 4 replicates (C) Intracellular triglyceride (Tg) amount determined by HPLC–ELSD (*P* < 0.0001). (D) Diacylglycerol acyltransferase (DGAT) mRNA expression (AU). *P < 0.05. (E) Fatty acid (FA) amount in cells (μg/10^6^ cell) analyzed by GC. (F) Membrane phospholipid (PL) concentration (μg/10^6^ cell) analyzed by HPLC-ELSD (G) PL membrane composition (weight %) analyzed by HPLC-ELSD n = 4 for each replicate in each treatment, total of 3 replicates.

Treatment with FFA increased the total number of LD in the cells (Fig 1A and 1B) compared to VLDL and controls. However, while the number of small LD increased 7-fold (*P* < 0.0001), the number of large ones increased 12-fold (*P* = 0.0009).

### FFA treatment increases Tg content and synthesis

**Fig 2:**
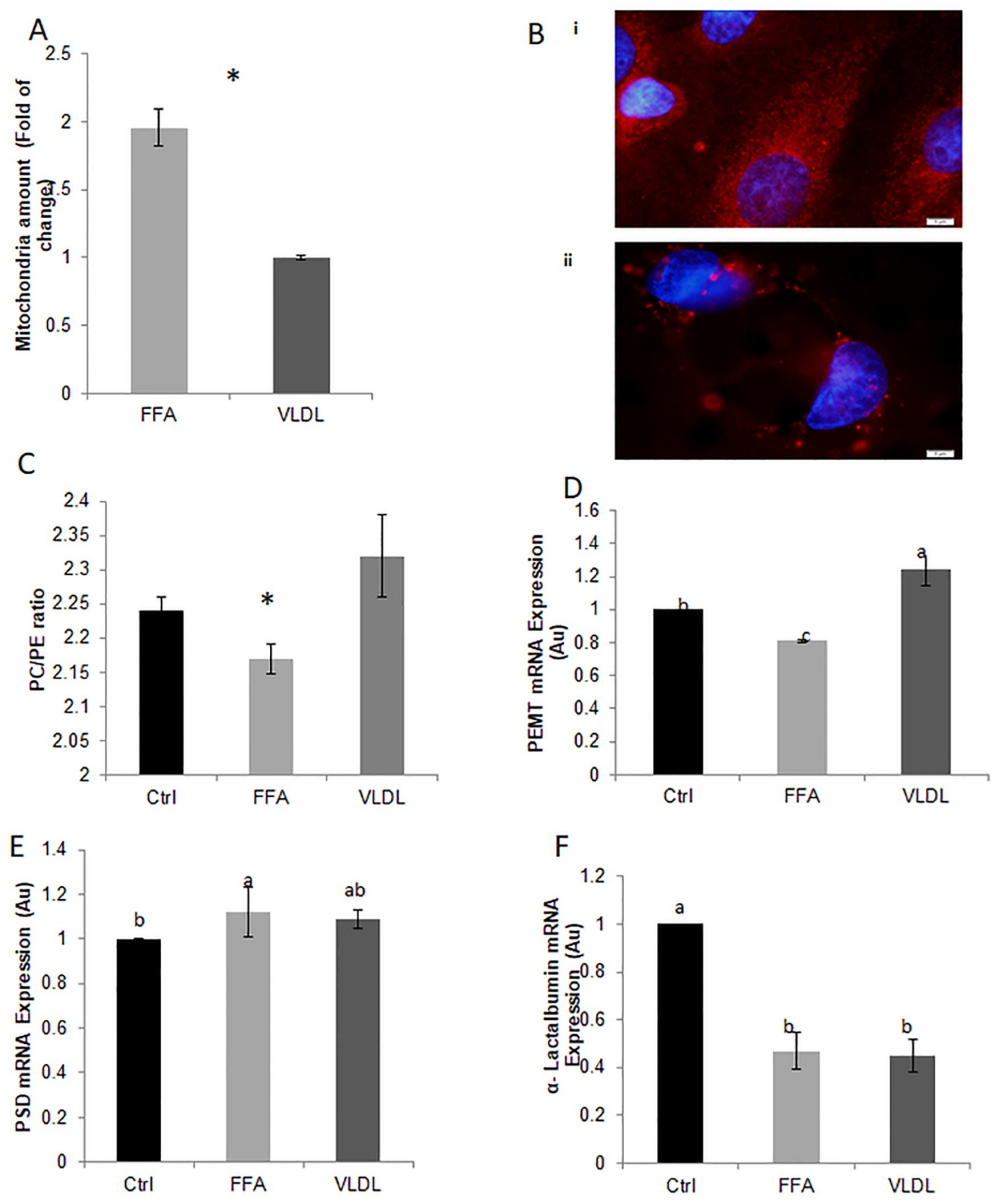
Long chain fatty acid macrostructure in culture medium alters mitochondrial quantity and gene expression of enzymes involved in the PL synthesis pathways in MEC. (A) Mitochondria was quantified by monitoring the fluorescence intensity of Mitotracker deep red staining and presented as fold change compared to the control. (B) Representative images of mitochondria amount in MEC treated with (i)FFA and (ii) VLDL. Scale bars, 5 μm n = 4 for each replicate in each treatment, total of 2 replicates (C) Ratio of phosphatidylcholine to phosphatidylethanolamine (PC/PE) determined by HPLC-ELSD (D) Phosphatidylethanolamine N-methyltransferase (PEMT) mRNA expression (AU) (E) Phosphatidylserine decarboxylase (PSD) mRNA expression (AU). (F) α-Lactalbumin mRNA expression (AU) n = 4 for each replicate in each treatment, total of 2 replicates.

The elevated number of LD in the FFA treatment was accompanied by a 3-fold increase in cellular Tg content compared to the VLDL treatment and controls (*P* < 0.0001, Fig 1C). The final step in Tg biosynthesis is catalyzed by acyl-CoA:diacylglycerol acyltransferase (DGAT) [50] and therefore the expression level of DGAT was determined. In accordance with the elevated Tg cellular content, *DGAT* mRNA expression was 2-fold higher in the FFA treatment (*P* < 0.05, Fig 1D). Higher Tg synthesis implies more fat production, which is related to MFG size. In addition, the *α-lactalbumin* mRNA expression was determined to assess whether the changes in MFG diameter and composition are related to the general cell productivity potential. *α-Lactalbumin* mRNA expression did not differ between FFA and VLDL treatments (Fig 2F).

### FA biochemical structure affects their availability to the cells

The cellular content of the exogenously administrated fatty acids (i.e. C16:0, C18:0, C18:1, C18:2) was determined. The results shows that the concentration of linoleic acid (C18:2) was 6 fold higher in cells treated with FFA compared to VLDL, however this difference was not significant due to large variability between repetitions (*P* < 0.1, Fig 1E). Oleic acid concentrations were higher in control vs. FFA and VLDL treatments (p = 0.0128). Palmitic acid in also tended to be higher in Ctrl compare to FFA and VLDL (p = 0.06).

### Membrane PL composition and LD size are modulated by exogenous long chain fatty acids macrostructure

Membrane PL concentration and composition differed between treatments (Fig 1F and 1G, respectively). The amount of PE, PS and PC were elevated by FFA treatment (*P* = 0.04, Fig 1F). However, when membrane composition was determined, PS content was reduced (*P* = 0.03) while that of PE tended to increase (*P* = 0.1, Fig 1G) in the FFA compared to controls and VLDL treatment. The reciprocal changes in PE and PS might be attributed to the 2 fold increase in the amount of mitochondria, in the FFA vs. VLDL treatment (*P* = 0.014, Fig 2A and 2B). The change in membrane PL concentration resulted in a lower PC/PE ratio for the FFA treatment (Fig 2C), suggesting a less stable membrane that is more prone to fusion.

Accordingly, LD size was larger under FFA treatment. The mRNA expression of two enzymes in the PL-synthesis pathway was examined, *PSD* and *PEMT*. *PEMT* mRNA expression was lower in the FFA vs. VLDL treatment and controls (*P* < 0.0009, Fig 2D). This suggests less PC synthesis and therefore a less stable membrane. In addition we determined the gene expression level of *PSD*, which catalyzes the PS to PE conversion in the mitochondria [25]. *PSD* mRNA expression was higher in FFA treatment vs. controls, but did not differ from the VLDL treatment (Fig 2E).

### Expression of genes related to fat and energy metabolism

**Fig 3:**
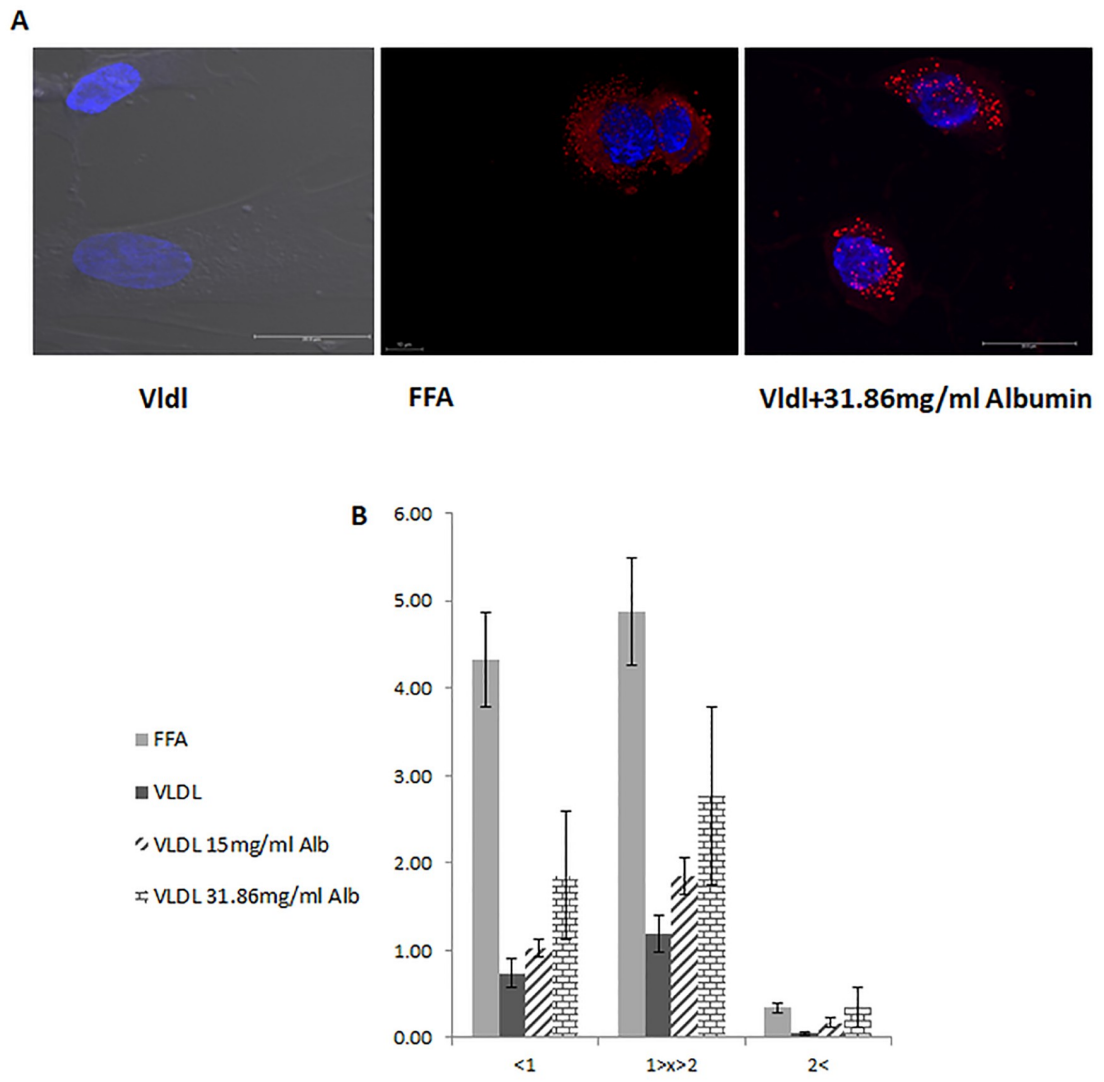
Albumin addition to MEC treated with VLDL result in similar response to that of FFA treated cells in terms of LD morphometric features. Mammary epithelial cells (MEC) treated with 100 μM VLDL, similar concentration and composition of fatty acids as FFA, or 100 μM VLDL with 15 mg/ml or 31.86 mg/ml albumin for 24 h. (A) Representative images of lipid droplets (LD) (scale bar 20 μm) in the various treatments. LD were more numerous and larger with FFA (i) compared to VLDL (ii). Albumin supplementation to VLDL treatment (iii) produced a LD phenotype similar to that with the FFA treatment. (B) Number of lipid droplets (LD) according to their size (<1 μm, 1–2 μm, >2 μm) in mammary epithelial cells (MEC) treated with VLDL (dark gray), FFA (light gray), 100 μM VLDL with 15 mg/ml albumin (wide upward diagonal), or 100 μM VLDL with 31.86 mg/ml albumin (horizontal brick) for 24h. n = 4 for each replicate in each treatment, total of 2 replicates.

*LPL* gene expression was lower in both FFA and VLDL treatments compared to controls (*P* = 0.0003, S1A Fig). To estimate if the limiting factor on VLDL-Tg hydrolysis is an acceptor of their product, albumin was added to the culture medium. A combined treatment with VLDL and albumin increased the number of small and large LD, resulting in a LD size phenotype which is more similar to that of the FFA-treated cells, in a concentration-dependent manner (Fig 3). *Ndufaf3* transcription levels were determined to evaluate the treatments effects on mitochondria content in the cell. In the mouse mammary gland, NDUFAF3 expression is elevated postpartum, concomitantly with increased mitochondria activity [20]. *Ndufaf3* mRNA expression did not differ between treatments (S1B Fig). Nevertheless, mRNA expression of *PPAR-γ*, which can promote mitochondria biogenesis, was 2 fold lower in the VLDL treatment compared to the FFA treatment and controls (S1C Fig). FABP transcription levels were numerically higher in FFA treated cells vs. VLDL (S2 Fig).

## Discussion

In the present study we provide first evidence for the role played by macrostructure of preformed long chain fatty acid (i.e. FFA vs. VLDL) in the regulation of LD morphometric features in MEC. These findings imply that the form of presentation of long chain fatty acids to MEC, is as an additional metabolic factor involved in the modulation of LD size, beyond the established role played by fatty acid composition and concentration in LD biosynthesis and MFG secretion.

### Increased fatty acids availability results in increased fat synthesis

MEC treated with FFA had higher concentration of cellular linoleic acid compared with cells treated with similar concentration and composition of fatty acids, in VLDL structure. Since linoleic acid originate from external source and cannot be synthesized endogenously in the cell, its higher cellular concentration indicates greater availability of exogenous, long chain fatty acids in this treatment. In addition, cells treated with FFA had higher Tg content accompanied by higher DGAT gene expression. DGAT catalyzes the final step of the biosynthesis of Tg [50]. Therefore its elevated expression may protect against lipotoxicity which may result from the higher availability of long chain fatty acids once administrated as FFA. This assumption is supported by the previously reported increase in cytosolic Tg accumulation in MEC treated with exogenous long-chain fatty acids in a concentration-dependent manner [28, 51–52]. The increased Tg content in cells treated with FFA was accompanied by a greater number of LD (Fig 1C and 1B). This is in agreement with the demonstration of a similar association between intracellular Tg content and LD number in cultured Drosophila s2 cells [53] and in cultured human hepatocytes [54].

It should be noted that LD size is usually positively correlated with cellular Tg content and lipogenesis capacity [55–57]. Similarly, a general positive association is often reported for MFG size and total milk fat concentration. This increased total milk fat content is often caused by reduced total milk yield. However, in the present study, no change was found in α-lactalbumin gene expression, a common proxy for milk synthesis capacity. Therefore, these results suggest that the effect of the macro-structure of long chain FA was not general and was focused on the lipid metabolic pathways which are involved in the modulation of LD size in MEC.

### Membrane composition and stability

Membrane stability, as determined by its PL composition, can modulate LD size in various cell types, including MEC [27–28]. In this study, higher PE and a tendency toward higher PC contents were found in VLDL and FFA treatments compared to controls. Nevertheless, the PC/PE ratio was lower in the FFA treatment, suggesting a less stable membrane [24] and more LD fusion [27]. Therefore, the higher percentage of large LD in the FFA treatment as demonstrated herein, is most probably attribute to the combination of higher Tg synthesis and greater fusion between LD. This will collectively increase the volume of fat (i.e. Tg) in the LD core, and will increase the LD size by promoting fusion between smaller LD. These results are in accordance with Cohen et al [28], who demonstrated that exposing MEC to free oleic acid induced the biosynthesis of larger LD attributed to greater fusion and elevated Tg content.

Changes in PL composition could be attributed to mitochondria function or number because of at least one of the conversion steps between PS to PE is executed by a mitochondria enzyme, PSD [25]. Here, we demonstrate higher number of mitochondria in the FFA treated cells (Fig 2A and 2B), which was associated with lower PS content in the membrane (Fig 1E). The reduced PPAR-γ expression in the VLDL compared with the FFA treatment (S1C Fig) support the hypothesis that mitochondria are involved in this process. The differences between treatments are in agreement with previous studies demonstrating that FFA induce mitochondrial biogenesis, through the PPAR-γ coactivator pathway [27,58].

### FFA acceptor reduces negative feedback on LPL activity and increase lipid droplet size

In the present study, upon exposure to VLDL or FFA, a reduction in LPL expression was recorded. This could be attributed to negative feedback of FFA, which were exogenously added to the culture medium or formed during VLDL hydrolysis. This assumption is in accordance with the findings of Kadegowda et al. [52] who found decreased LPL expression in MEC treated with free palmitic acid, stearic acid and oleic acid.

The released products of VLDL hydrolysis can allosterically inhibit LPL activity [59]. LPL activity requires a product acceptor, such as BSA or calcium ions, to catalyze the in-vitro hydrolysis of VLDL- Tg [60]. As the fatty acids-binding sites on BSA become filled with product, the rate of Tg hydrolysis slows down [60] and some of the enzymatic activity will be sequestered. This might explain why, in this study, a higher concentration of albumin combined with VLDL, resulted in a similar phenotype as that received in MEC treated with FFA (Fig 3A). The absorption process of FFA requires fatty acid binding proteins (FABP) which play an important role in the fatty acids transport between the different compartments of the cell. The higher numerical difference found in FABP transcription levels in FFA treated cells vs. VLDL (S2 Fig) is in accordance with previous studies in hepatocytes and adipocytes exposed to elevated extracellular lipid levels [61].

This work shows for the first time that the macrostructure of long chain fatty acids available to MEC has a role in regulating LD phenotype in terms of number and diameter, as well as the lipid composition of the cells and its membranes. In addition, the results suggest that LD size is modulated by the availability of long chain FA from the extracellular fluid, and not exclusively by total fat concentration and milk and fat synthesis capacity of the cells, as often assumed in in vitro and in vivo models of mammary gland and other lipogenic tissues.

## Supporting information

S1 Fig Mammary epithelial cells (MEC) treated with 100 μM VLDL or similar concentration and composition of for 24 h. (A) Lipoprotein lipase (LPL) mRNA expression (AU). (B) NADH dehydrogenase (ubiquinone) 1α subcomplex assembly factor 3 (NDUFAF3) mRNA expression (AU) (C) Peroxisome proliferator-activated receptor gamma (PPAR-γ) mRNA expression (AU).
(TIF)

S2 Fig Mammary epithelial cells (MEC) treated with 100 μM VLDL or similar concentration and composition of for 24 h. Fatty Acid Binding Protein (FABP) mRNA expression (AU).
(TIF)

S1 Data Data for all the result are presented as mean ±SE ans SEM. (XLSX)
