# Supplementary figures and images for: Changes in lipid droplets morphometric features in mammary epithelial cells upon exposure to non-esterified free fatty acids compared with VLDL
Source: PLoS One. 2018 Dec 31;13(12):e0209565. doi: 10.1371/journal.pone.0209565 (PMC6312266; doi:10.1371/journal.pone.0209565)

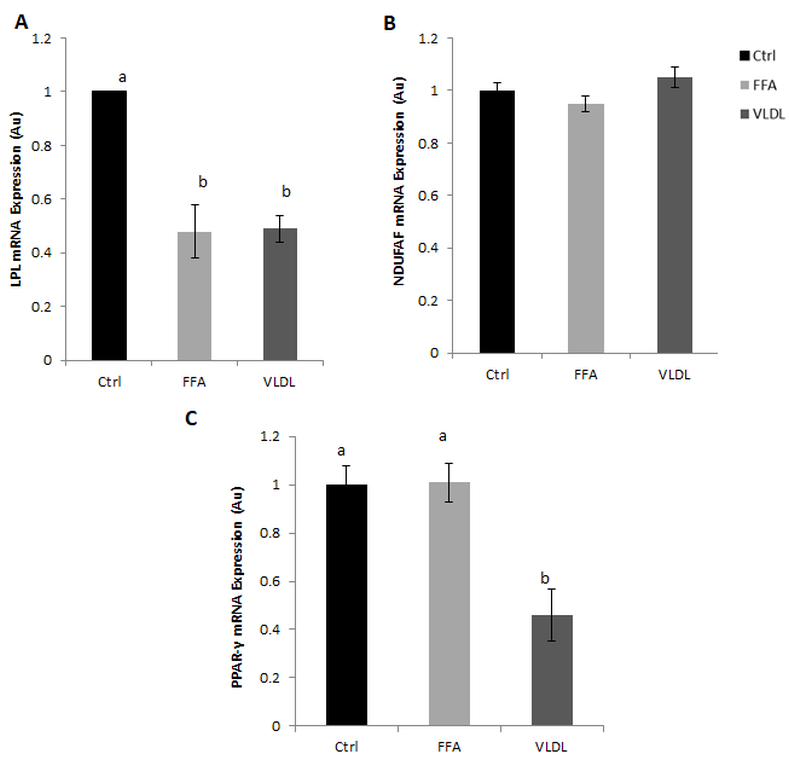

Supplement: S1 Fig — (A) Lipoprotein lipase (LPL) mRNA expression (AU). (B) NADH dehydrogenase (ubiquinone) 1α subcomplex assembly factor 3 (NDUFAF3) mRNA expression (AU) (C) Peroxisome proliferator-activated receptor gamma (PPAR-γ) mRNA expression (AU). (TIF) [file pone.0209565.s001.tif]

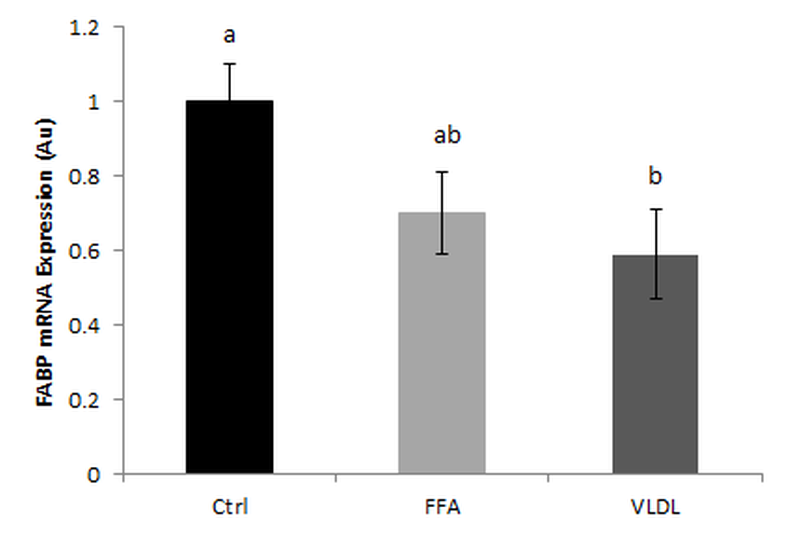

Supplement: S2 Fig — Fatty Acid Binding Protein (FABP) mRNA expression (AU). (TIF) [file pone.0209565.s002.tif]
